# Comparison of self-reported and accelerometer-assessed measurements of physical activity according to socio-demographic characteristics in Korean adults

**DOI:** 10.4178/epih.e2018060

**Published:** 2018-11-29

**Authors:** Seung Won Lee, Jee-Seon Shim, Bo Mi Song, Ho Jae Lee, Hye Yoon Bae, Ji Hye Park, Hye Rin Choi, Jae Won Yang, Ji Eun Heo, So Mi Jemma Cho, Ga Bin Lee, Diana Huanan Hidalgo, Tae-Hoon Kim, Kyung Soo Chung, Hyeon Chang Kim

**Affiliations:** 1 Department of Public Health, Yonsei University of Graduate School, Seoul, Korea; 2 Cardiovascular and Metabolic Diseases Etiology Research Center, Yonsei University College of Medicine, Seoul, Korea; 3 Department of Preventive Medicine, Yonsei University College of Medicine, Seoul, Korea; 4 Department of Cardiology, Severance Cardiovascular Hospital, Yonsei University College of Medicine, Seoul, Korea; 5 Department of Internal Medicine, Institute of Chest Disease, Yonsei University College of Medicine, Seoul, Korea

**Keywords:** Physical activity, Accelerometer, Questionnaire, Korea

## Abstract

**OBJECTIVES:**

Previous studies have shown relatively low correlations between self-reported and accelerometer-assessed physical activity (PA). However, this association differs by socio-demographic factors, and this relationship has not been fully investigated in the general population. Thus, we investigated the correlation between self-reported and accelerometer-assessed PA and whether it differed by demographic and socioeconomic factors among the Korean general population.

**METHODS:**

This cross-sectional study included 623 participants (203 men and 420 women) aged 30 to 64 years, who completed a PA questionnaire and wore a wrist-worn accelerometer on the non-dominant wrist for 7 days. We examined the agreement for metabolic equivalent task minutes per week (MET-min/wk) between the 2 measures and calculated Spearman correlation coefficients according to demographic and socioeconomic factors.

**RESULTS:**

The kappa coefficient between tertiles of self-reported and accelerometer-assessed total MET-min/wk was 0.16 in the total population, suggesting overall poor agreement. The correlation coefficient between the 2 measurements was 0.26 (p<0.001) in the total population, and the correlation tended to decrease with increasing age (p for trend <0.001) and depression scores (p for trend <0.001).

**CONCLUSIONS:**

We found a low correlation between self-reported and accelerometer-assessed PA among healthy Korean adults, and the correlation decreased with age and depression score. When studying PA using accelerometers and/or questionnaires, age and depression need to be considered, as should differences between self-reported and accelerometer-assessed PA.

## INTRODUCTION

Physical activity (PA) is an important modifiable risk factor for cardiovascular disease (CVD), diabetes mellitus, falls, osteoporosis, obesity, some cancers, and mortality [1-4]. Epidemiologic studies usually assess PA by self-reported questionnaires for practical reasons [5]. However, self-reported data are vulnerable to reporting bias [6]. Moreover, light-intensity activities are hard to recall and tend to be under-reported [7,8]. These errors in the measurement of PA might attenuate estimates of the effect of PA on health-related outcomes [9]. Objective measures, such as pedometers and accelerometers, have emerged as an alternative to solve these problems. Accelerometers can provide objective estimates of the duration and intensity of PA [5,10]. Agreement between questionnaire- and accelerometer-assessed PA was remarkably low in previous studies [5,11]. Recall and response bias in questionnaire surveys might be largely responsible for discrepancies between these 2 measures [12]. Previous studies reported that these biases can be influenced by demographic factors, socioeconomic status, and health status [13,14]. Furthermore, accelerometers attached to the upper body cannot detect certain activities that only use the lower body, such as weightlifting and cycling. Previous studies also reported that the association between questionnaire- and accelerometer-assessed PA differed by age, gender, ethnicity, socioeconomic status, and level of PA [5,13,14]. However, there are limited data on the association between questionnaire- and accelerometer-assessed levels of PA in the Korean population. Thus, we compared questionnaire-based and accelerometer-assessed PA among Korean adults, and investigated whether socio-demographic factors affected the correlation between these 2 measurements of PA.

## MATERIALS AND METHODS

### Study population

This study was conducted utilizing baseline data from the Cardiovascular and Metabolic Diseases Etiology Research Center (CMERC) study, which was launched in 2013. The CMERC study consists of 2 prospective cohorts: a general population cohort (the CMERC cohort) and a cohort of high-risk patients (the CMERC-HI cohort). The data collection procedures of the CMERC cohort have been described elsewhere in detail [15]. Wrist-worn accelerometry was performed in a subsample of the participants in the CMERC cohort operated by the Department of Preventive Medicine, Yonsei University College of Medicine. They were relatively healthy people without a history of major CVD, such as myocardial infarction or stroke, when they were enrolled in the CMERC cohort.

Between December 2013 and September 2017, a total of 738 individuals participated in PA measurements using a 3-dimensional accelerometer. They all completed health questionnaires and health examinations using an identical protocol. In the current study, participants were included if they had available PA data from the accelerometer for at least 16 hr/d for 7 days. After excluding 101 persons with invalid accelerometer data and 4 persons with unreliable accelerometer data, 623 participants (203 men and 420 women) aged 30 to 64 years old were included in the current analysis. All participants provided written informed consent, and the institutional review board of Severance Hospital, Yonsei University Health System, Seoul, Korea (4-2013-0661) approved the study protocol.

### Measurement of physical activity by questionnaire

For the questionnaire-based assessment of PA, we used a Korean version of the International Physical Activity Questionnaire (IPAQ)-Short Form, which asks for the frequency of each activity and the duration thereof during the past 7 days [16]. The short form records activities at 4 intensity levels: (1) vigorous-intensity activity such as aerobics, (2) moderate-intensity activity such as leisure cycling, (3) walking, and (4) sitting. According to the IPAQ scoring protocol [17], participants’ responses were converted to metabolic equivalent task minutes per week (MET-min/wk). Using the Ainsworth et al. [18] compendium, an average MET score was derived for each type of activity [18]. The following values were used for the analysis of IPAQ data: walking=3.3 METs, moderate PA=4.0 METs, vigorous PA=8.0 METs, and total PA MET-min/wk=sum of walking+moderate +vigorous MET-min/wk scores. A previous study reported Spearman rho coefficients and kappa values of test-retest reliability in Korean adults aged 15-69 years of 0.427-0.646 (median, 0.542) and 0.365-0.620 (median, 0.471), respectively [19]. The kappa values were greater than 0.4 in 5 of the 7 questionnaires. In a study of elderly individuals, the Spearman rho coefficients and kappa values of test-retest reliability for 5 parameters (vigorous days, vigorous minutes, moderate days, moderate minutes, and walk days) were 0.299-0.605 and 0.307-0.418, respectively [16].

### Measurement of physical activity by accelerometer

For the accelerometer-based assessment of PA, a wrist-worn tri-axial accelerometer (GENEActiv; Activinsights Ltd., Kimbolton, UK) was used. The accelerometers were pre-programmed with a 100-Hz sampling frequency and participants were asked to wear the accelerometer on their non-dominant wrist for 7 consecutive days and nights. The raw data were downloaded to a personal computer using the software supplied by the manufacturer (GENEActiv version 2.2) and transformed into 1-minute epoch files. To obtain values including the duration of each activity and MET score for the current analyses, we used the GENEActiv macro file ‘General physical activity’ version 1.8, which was previously validated [20,21]. All participants continued to wear accelerometers at night.

### Other questionnaire data

The CMERC cohort study collected demographic and socioeconomic data on gender, age, education, marital status, and household income [15]. Marital status was defined as living with a partner or not. Education was categorized as primary school or below, lower secondary school, higher secondary school or university degree or higher. Income level was categorized as lower, middle, or upper based on tertile values of annual household income.

Cognitive function was only assessed in participants aged 50 years or older, using the Korean version of the Mini-Mental State Estimation (MMSE) for dementia screening [22]. MMSE scores range from 0 to 30, with a higher score indicating better cognitive performance. We used a cutoff of 26 to categorize participants as having cognitive impairment, as in previous studies [13,23]. Depressive symptoms were assessed using the Korean version of the Beck Depression Inventory-II (BDI) [24,25].

### Anthropometric measurements

Standing height was measured to the nearest 0.1 cm using a stadiometer (DS-102, Jenix, Seoul, Korea). Body weight was measured to the nearest 0.1 kg on a digital scale (DB-150, CAS, Seongnam, Korea) according to a predefined protocol [15]. Body mass index (BMI) was calculated as an individual’s body weight in kilograms divided by his or her height in meters squared.

### Statistical analyses

Gender differences were analyzed using the independent *t*-test or the Wilcoxon rank-sum test for continuous variables and the chi-square test for categorical variables. In order to investigate agreement between PA (MET-min/wk) measured by the questionnaire and the accelerometer, we compared tertile values for the 2 measurements using the kappa index. The correlation between questionnaire and accelerometer-assessed PA was evaluated using Spearman correlation coefficients, along with Bland-Altman plots. These analyses were conducted for the total population, and then separately for the following categories: gender, age group (i.e., 30-39, 40-49, 50-59, and ≥60 years), BMI category, marital status, education, household income, cognitive function, and prevalent depression.

Since the Spearman correlation coefficient is equal to the slope of the regression between the ranked values of the 2 measures, gender differences were tested by regressing the gender-specific rank of accelerometer-assessed total MET-min/wk on the gender-specific rank of questionnaire-assessed total MET-min/wk together with the interaction term (gender×rank of questionnaire-assessed PAs) using a linear model, similarly to a previous study [14]. The p-value for interaction was used to test whether the correlation between questionnaire-based and accelerometer-assessed PA differed by gender. This analysis was repeated for the demographic and socioeconomic variables under consideration. For age, BMI, educational level, income, and BDI score, the p-value for the trend across categories was also calculated by fitting a linear group interaction term with the rank of MET-min/wk. All analyses were performed using SAS version 9.4 (SAS Institute Inc., Cary, NC, USA). All statistical tests were 2-sided and p-values less than 0.05 were considered to indicate statistical significance.

## RESULTS

The general characteristics of the study population are presented in Table 1. The mean age was 52.5 years in men and 53.3 years in women. The median (interquartile range) of total MET-min/wk was 1,590 (693-3,228) when measured by the questionnaire and 12,457 (11,053-14,044) when measured by the accelerometer. Overall, PA levels measured by the questionnaire were lower than those measured by the accelerometer. With borderline significance, the total MET-min/wk measured by the questionnaire was higher in men than in women, but the total MET-min/wk measured by the accelerometer was higher in women than in men.

**Figure 1.**
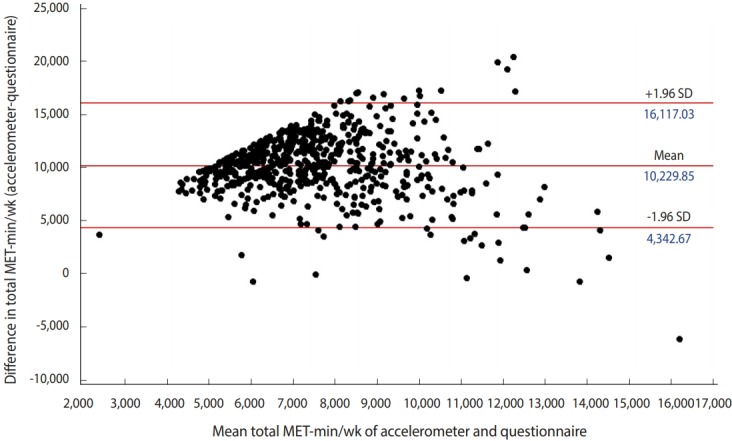
Bland-Altman plot for metabolic equivalent task minutes per week (MET-min/wk) assessed by a questionnaire and an accelerometer. Horizontal lines are drawn at the mean difference in total MET-min/wk, and at the limits of agreement. SD, standard deviation.

Table 2 shows the cross-classification of tertile groups of self-reported and accelerometer-assessed total MET-min/wk. The kappa coefficient was 0.16 in men and 0.19 in women, suggesting overall poor agreement. The strength of agreement between self-reported and accelerometer-assessed MET-min/wk is shown in Figure 1.

Table 3 shows correlations between the questionnaire and the accelerometer when measuring the time participants spent engaged in different types of PA and MET-min/wk. In total population, the correlation coefficient between self-reported sitting time and accelerometer-assessed sedentary time was 0.36 (p<0.001), which was the highest correlation coefficient observed in the current study. The correlation coefficient between self-reported and accelerometer-assessed time was 0.20 (p<0.001) for vigorous activity and 0.19 (p<0.001) for moderate-intensity activity.

The correlation coefficient between self-reported and accelerometer-assessed MET-min/wk was 0.26 (p<0.001) in the total population (Table 4). The correlation did not significantly differ by gender, age, BMI, marital status, education, income, cognitive function, or depression. However, as age and depression scores increased, the correlation between self-reported and accelerometer-assessed PA tended to decrease (p for trend in age and depression score <0.001, respectively). Additionally, when we investigated correlations between questionnaire- and accelerometer-assessed PA according to occupation (white collar, blue collar, and unemployed), no significant differences in the strength of the correlation among occupational groups were found (data not shown).

## DISCUSSION

We examined the relationship between self-reported and accelerometer-assessed PA and whether the relationship differed by demographic and socioeconomic factors. Overall, less PA was measured by the questionnaire than by the accelerometer. This is probably because activities of short duration, for instance lasting less than 10 minutes, are unlikely to be captured by a questionnaire, but can be detected by an accelerometer. The mean time of vigorous activity in our study population was lower than in previous studies, but the total PA level (MET-min/wk) was similar [26]. It is possible that the participants responded incorrectly to questionnaire items about exercise intensity.

The overall correlation between self-reported and accelerometer-assessed PA in our study (total MET-min/wk, r=0.26) was relatively low, and the correlation decreased with increasing age and depression score. A significant difference was found in PA according to season, but there was no significant seasonal effect on the correlation between the 2 measurements (data not shown).

The correlation between questionnaire- and accelerometer-assessed PA in our study was similar to the results of previous studies. In a previous study with 1,270 Hong Kong Chinese participants, the overall Spearman correlation between IPAQ-assessed and accelerometer-assessed PA (MET-min/wk) ranged from 0.06 to 0.24 [27]. The Spearman correlation coefficient between questionnaire- and accelerometer-assessed PA was 0.33 (95% confidence interval [CI], 0.30 to 0.36) in the Whitehall II study and 0.30 (95% CI, 0.25 to 0.34) in the Rotterdam study [13,14]. The Whitehall ΙΙ study also reported that the correlation between the 2 measurements was higher for more energetic activities [14]. However, our data showed that the highest correlation was found between questionnaire-assessed sitting time and accelerometer-assessed sedentary activity time. The discrepancy between our results and those of that previous study may have been due to the use of a different type of questionnaires (the IPAQ vs. the Minnesota Leisure Time Physical Activity Questionnaire [28,29]) and differences in the characteristics of the study populations.

Regarding the influence of demographic and socioeconomic factors on the correlation between questionnaire- and accelerometer-assessed PA, most previous studies showed a higher correlation in men [12,16,30-32], younger people [27,30-32], and those with higher levels of education [14,27].

A study with Hong Kong Chinese participants reported that gender, age, job status (full-time worker or not), educational level, and obesity could influence the validity of the IPAQ, but did not appear to influence the correlation between IPAQ and accelerometer data [27]. In the Whitehall ΙΙ study, the correlation was higher in people with a high educational level or occupational position than in people with a low educational level or occupational position [14]. In the Rotterdam study, people with high education had a greater correlation coefficient, and people with obesity, a higher disability score, and more depressive symptoms had a greater difference in the 2 measures [13].

In our data, older people and those with a higher depression score tended to have lower correlation coefficients than their younger or healthier counterparts. However, the correlation between questionnaire- and accelerometer-assessed PA did not differ by gender, marital status, household income, or MMSE score. The questionnaire survey showed that the oldest age group (aged over 60 years) had the highest level of PA (MET-min/wk), while the accelerometer test showed the lowest level of PA in the oldest age group (data not shown).

The possible reasons why the correlation between self-reported and questionnaire-assessed PA decreased as age increased include memory difficulties and cognitive problems, which are more prevalent in elderly adults. The questions of the IPAQ-Short Form that require the use of recognition memory are preferred over those that require recall [33,34]. However, in our study, cognitive function, as assessed by the MMSE score, was not associated with the correlation between self-reported and questionnaire-assessed PA. Additionally, there were few people who had cognitive problems in our study. Another possibility is that the open-ended response format of the IPAQ-Short Form can be difficult for elderly adults to complete accurately [35].

A previous study reported that the measurement method of PA is important when investigating associations between PA and depression [36]. Depressed persons show a response bias favoring the reporting of negative self-relevant information [37]. Reporting bias may therefore have influenced the current study.

Accelerometers have been often used in validation studies [12,32], but they are not a gold standard, since they measure the movement of only a single part of the body, but the resulting inferences are applied to the whole body. In addition, previous studies have proposed thresholds to define mild, moderate, and vigorous levels of PA and developed algorithms for detecting various types of PA [21,38,39]. However, there is no consensus on the best method, and considerable inconsistencies exist in results derived from different algorithms [14]. Measuring PA by questionnaire is the most cost-effective method, and questionnaires can be used to assess all types of PA and in large populations in epidemiological research [5]. Questionnaires can also assess PA for a relatively long time period. However, self-reported PA by questionnaire has several limitations, such as reporting and recall bias, as well as the inability to capture the absolute level of PA [12]. Activities of light intensity are hard to recall and might not be reported [7,13]. Furthermore, moderate or vigorous activities performed for a very short duration might not be recalled by the participants when they respond to the questionnaire [13]. Particular caution must be taken when using a questionnaire for young and elderly participants, as their memory can be incomplete [40,41]. In particular, older adults are more likely to engage in light- to moderate-intensity PA, which is the most difficult type of activity to assess through a questionnaire [42]. In addition, the IPAQ only includes activities of moderate or vigorous intensity carried out for more than an hour, which may explain the underestimation of PA in the questionnaire [13]. To redeem the limitations of the questionnaire, researchers have used motion sensors, such as pedometers or accelerometers, as an additional measurement for assessing PA in a free-living environment [43]. Accelerometers can record the acceleration associated with body movement, which can provide information on the duration and intensity of certain PAs [44]. Accelerometers include all PAs, including small bouts of activity (less than 5 minutes) and can avoid recall and response bias. Despite the advantages of using accelerometers, they are time-consuming and costly to apply in studies with a large-scale epidemiological research design. Additionally, PA measured with a wrist-worn accelerometer can be underestimated when an individual engages in PA with the wrist fixed, such as carrying a briefcase, or PA that only involves the legs, such as cycling [45]. Additionally, when collecting PA data using an accelerometer, caution should be taken regarding variation in participants’ compliance in terms of wearing the device and seasonal variation reflecting the possibility of water-based activities [5]. Because both accelerometers and questionnaires have advantages and disadvantages, using both measures is recommended as a way to collectively measure an individual’s PA. Further studies are also required to develop a better understanding of the association between questionnaire- and accelerometer-assessed PA.

Our study contains several strengths. First, we used a validated accelerometer and questionnaire; thus, our results can be compared with previous studies that used the same assessment tools. Second, our study showed relatively high compliance for accelerometer wearing. Third, our study population consisted of a large number of community-dwelling adults from a large population-based cohort.

However, our study also had some limitations. First, the PA data derived from the accelerometer and questionnaire were not obtained in the same week. The accelerometer measurements were made over a 7-day period after participants completed the questionnaire. This might have contributed to the low correlation coefficients between self-reported and accelerometer-assessed PA in the current study. Second, as the data were drawn from a subsample of the CMERC cohort, which consists of community-dwelling healthy people aged 30-65 years without a history of CVD, who might have had a different PA pattern from those who are less active. Furthermore, those who were extremely active might have felt too much pressure from the accelerometer and refused to participate because the accelerometer could interfere with their activity and there was a risk that the device would break during PA [27]. However, in our sensitivity analysis, there were no significant differences in characteristics such as gender, marital status, education level, depression score, BMI, and blood pressure between people who participated in the accelerometer component of the study and those who refused to participate (data not shown). However, the mean age and MMSE scores of those who chose to participate in the accelerometer component were higher than those of individuals who did not participate. A third limitation is the lack of randomization. However, our study utilized a community-based cohort design, which represents real-world circumstances well [46]. Fourth, we used a wrist-worn accelerometer due to expected higher compliance [47]. Previous studies have typically used hip-worn accelerometers in order to better reflect lower body movements [47,48]. The National Health and Nutrition Examination Survey, which conducts surveillance of PA in the US population, previously used a uniaxial accelerometer worn on the hip (2003–2004 and 2005–2006), but changed its protocol and asked participants to wear a triaxial accelerometer on the wrist during recent surveys (2011–2014) among persons aged over 6 years [47,49]. Also, several studies reported that hip and wrist-worn accelerometers were moderately correlated in adults and adolescents [49,50]. Finally, although our results are in accordance with those of previous studies that used different instruments and a different type of accelerometer, our results might not be generalizable to other instruments.

In conclusion, we found a low correlation between self-reported and accelerometer-assessed PA among healthy Korean adults, and the correlation decreased with age and depression score. Future studies assessing PA using questionnaires and/or accelerometers should take these results into account.

## Figures and Tables

**Table 1.** General characteristics of the study population

Variables	Total (n=623)	Men (n=203)	Women (n=420)	p-value^1^
Age (yr)	53.0±9.1	52.5±10.2	53.3±8.6	0.31
BMI (kg/m^2^)	23.8±2.9	24.5±2.7	23.5±3.0	<0.001
Marital status				
Married/cohabiting	544 (87.3)	195 (96.1)	349 (83.1)	<0.001
Single	79 (12.7)	8 (3.9)	71 (16.9)	
Education				
Secondary school or below	318 (51.0)	73 (36.0)	245 (58.3)	<0.001
University degree or more	305 (49.0)	130 (64.0)	175 (41.7)	
Income				
Lower	195 (31.3)	46 (22.7)	149 (35.5)	0.005
Middle	193 (31.0)	69 (34.0)	124 (29.5)	
Upper	235 (37.7)	88 (43.4)	147 (35.0)	
MMSE score				
<26	225 (36.1)	76 (37.4)	149 (35.5)	0.63
≥26	398 (63.9)	127 (62.6)	271 (64.5)	
BDI score				
None (0-13)	474 (76.1)	170 (83.7)	304 (72.4)	0.01
Mild (14-19)	93 (14.9)	24 (11.8)	69 (16.4)	
Moderate (20-28)	43 (6.9)	7 (3.5)	36 (8.6)	
Severe (29-63)	13 (2.1)	2 (1.0)	11 (2.6)	
Physical activity by questionnaire (min/wk)				
Sitting time	2,691±1,395	3,004±1,518.6	2,539±1,306	<0.001
Walking time	280 [120-600]	270 [120-525]	300 [120-600]	0.55
Moderate activity time	0 [0-140]	0 [0-180]	0 [0-120]	0.06
Vigorous activity time	0 [0-0]	0 [0-120]	0 [0-0]	<0.001
Total MET	1,590 [693-3,228]	1,782 [716-3,626]	1,560 [693-3,113]	0.08
People with moderate activity ≥ 60	45 (7.2)	21 (10.3)	24 (5.7)	0.05
People with vigorous activity ≥ 600	121 (19.4)	60 (29.6)	61 (14.5)	<0.001
Physical activity by accelerometer (min/wk)				
Sedentary activity time	4,605±791.4	4,860±822.1	4,482±747	<0.001
Light activity time	780 [601-964]	637 [493-896]	825 [694-999]	<0.001
Moderate activity time	1,129 [842-1,495]	996 [759-1,355]	1,175 [907-1,535]	<0.001
Vigorous activity time	28 [13-58]	40 [20-82]	23 [11-50]	<0.001
Total MET	12,457 [11,053-14,044]	12,211 [10,861-13,765]	12,595 [11,295-14,114]	0.07
People with moderate activity ≥ 60	570 (91.5)	179 (88.2)	391 (93.1)	0.06
People with vigorous activity ≥ 600	524 (84.1)	185 (9.1)	339 (80.7)	<0.001

Values are presented as mean ± standard deviation, median [interquartile range], or number (%). BMI, body mass index; MMSE, Mini-Mental State Estimation; BDI, Beck Depression Inventory-II; MET, metabolic equivalent task.

1 p-values were derived from the independent t-test, the Wilcoxon rank sum test, or chi-square test.

**Table 2.** Agreement between tertiles of self-reported and accelerometer-assessed total MET-min/wk

Self-reported MET-min/wk	Accelerometer-measured MET-min/wk			Kappa statistic
	Lower	Middle	Upper	
Total (n=623)				
Lower	91 (44.4)	61 (28.8)	49 (23.8)	0.16
Middle	67 (32.7)	80 (37.7)	69 (33.5)	
Upper	47 (22.9)	71 (33.5)	88 (42.7)	
Men (n=203)				
Lower	27 (40.9)	19 (27.1)	18 (26.9)	0.16
Middle	26 (39.4)	27 (38.6)	19 (28.4)	
Upper	13 (19.7)	24 (34.3)	30 (44.8)	
Women (n=420)				
Lower	63 (45.7)	43 (30.1)	30 (21.6)	0.19
Middle	45 (32.6)	53 (37.1)	47 (33.8)	
Upper	30 (21.7)	47 (32.9)	62 (44.6)	

MET, metabolic equivalent task.

**Table 3.** Correlations between self-reported and accelerometer-assessed PA per week

Accelerometer-measured PA (time)	Self-reported PA (time)									
	Sitting		Walking		Moderate activity		Vigorous activity		Total MET-min/wk	
	r	p-value	r	p-value	r	p-value	r	p-value	r	p-value
Total population (n=623)										
Sedentary activity	0.36	<0.001	-0.12	0.004	-0.11	0.006	0.03	0.43	-0.16	<0.001
Light activity	-0.30	<0.001	0.05	0.17	0.08	0.04	-0.07	0.10	0.06	0.17
Moderate activity	-0.33	<0.001	0.21	<0.001	0.19	<0.001	0.04	0.34	0.29	<0.001
Vigorous activity	-0.08	0.04	0.09	0.02	0.11	0.007	0.20	<0.001	0.22	<0.001
Total MET-min/wk	-0.29	<0.001	0.16	<0.001	0.18	<0.001	0.07	0.06	0.26	<0.001
Men (n=203)										
Sedentary activity	0.36	<0.001	-0.21	0.002	-0.19	0.008	-0.15	0.04	-0.35	<0.001
Light activity	-0.23	0.001	0.07	0.30	0.10	0.17	-0.02	0.79	0.12	0.10
Moderate activity	-0.28	<0.001	0.21	0.003	0.24	<0.001	0.16	0.02	0.37	<0.001
Vigorous activity	-0.12	0.09	0.02	0.77	0.05	0.46	0.15	0.03	0.12	0.09
Total MET-min/wk	-0.23	0.001	0.12	0.08	0.21	0.003	0.10	0.15	0.27	<0.001
Women (n=420)										
Sedentary activity	0.30	<0.001	-0.06	0.18	-0.11	0.02	0.06	0.20	-0.10	0.05
Light activity	-0.28	<0.001	0.01	0.77	0.11	0.02	<0.01	0.95	0.04	0.43
Moderate activity	-0.31	<0.001	0.20	<0.001	0.19	<0.001	0.02	0.68	0.27	<0.001
Vigorous activity	-0.12	0.02	0.14	0.003	0.12	0.01	0.17	<0.001	0.26	<0.001
Total MET-min/wk	-0.30	<0.001	0.17	<0.001	0.17	<0.001	0.09	0.06	0.26	<0.001

PA, physical activity; MET, metabolic equivalent task.

**Table 4.** Spearman correlation between self-reported and accelerometer-assessed physical activity according to demographic and socioeconomic factors

	n (%)	Total MET-min/wk			p for trend
		r	p-value	p for difference	
Total population	623 (100)	0.26	<0.001		
Gender					
Men	203 (32.6)	0.27	<0.001	0.90	N/A
Women	420 (67.4)	0.26	<0.001		
Age group (yr)					
30-39	83 (13.3)	0.31	0.004	0.29	<0.001
40-49	94 (15.1)	0.29	0.004		
50-59	268 (43.0)	0.31	<0.001		
60-64	178 (28.6)	0.13	0.09		
BMI (kg/m^2^)				0.10	0.37
<22.9	252 (40.4)	0.27	<0.001		
23.0-24.9	175 (28.1)	0.26	<0.001		
25.0-29.9	182 (29.2)	0.26	<0.001		
≥30.0	14 (2.3)	0.24	0.40		
Marital status					
Married/cohabiting	544 (87.3)	0.23	<0.001	0.80	N/A
Single	79 (12.7)	0.41	<0.001		
Education					
Secondary school or below	318 (51.0)	0.24	<0.001	0.81	N/A
University degree or more	305 (49.0)	0.26	<0.001		
Income					
Lower	195 (31.3)	0.27	<0.001	0.17	0.25
Middle	193 (31.0)	0.34	<0.001		
Upper	235 (37.7)	0.17	0.009		
MMSE score					
<26	225 (36.1)	0.33	<0.001	0.34	N/A
≥26	398 (63.9)	0.22	<0.001		
BDI score					
None (0-13)	474 (76.1)	0.26	<0.001	1.00	<0.001
Mild (14-19)	93 (14.9)	0.26	0.01		
Moderate (20-28)	43 (6.9)	0.21	0.17		
Severe (29-63)	13 (2.1)	- 0.23	0.45		

MET, metabolic equivalent task; BMI, body mass index; MSE, Mini-Mental State Estimation; BDI, Beck Depression Inventory-II; N/A, not applicable.
